# Loss of PTEN Is Not Associated with Poor Survival in Newly Diagnosed Glioblastoma Patients of the Temozolomide Era

**DOI:** 10.1371/journal.pone.0033684

**Published:** 2012-03-29

**Authors:** Christine Carico, Miriam Nuño, Debraj Mukherjee, Adam Elramsisy, Jocelynn Dantis, Jethro Hu, Jeremy Rudnick, John S. Yu, Keith L. Black, Serguei I. Bannykh, Chirag G. Patil

**Affiliations:** 1 Department of Neurosurgery, Center for Neurosurgical Outcomes Research, Maxine Dunitz Neurosurgical Institute, Cedars-Sinai Medical Center, Los Angeles, California, United States of America; 2 Department of Pathology, Division of Neuropathology, Cedars-Sinai Medical Center, Los Angeles, California, United States of America; The Chinese University of Hong Kong, Hong Kong

## Abstract

**Introduction:**

Pre-temozolomide studies demonstrated that loss of the tumor suppressor gene PTEN held independent prognostic significance in GBM patients. We investigated whether loss of PTEN predicted shorter survival in the temozolomide era. The role of PTEN in the PI3K/Akt pathway is also reviewed.

**Methods:**

Patients with histologically proven newly diagnosed GBM were identified from a retrospective database between 2007 and 2010. Cox proportional hazards analysis was used to calculate the independent effects of PTEN expression, age, extent of resection, Karnofsky performance scale (KPS), and treatment on overall survival.

**Results:**

Sixty-five percent of patients were men with median age of 63 years, and 70% had KPS≥80. Most patients (81%) received *standard* treatment (temozolomide with concurrent radiation). A total of 72 (47%) patients had retained PTEN expression. Median overall survival (OS) was 19.1 months (95% CI: 15.0–22.5). Median survival of 20.0 months (95% CI: 15.0–25.5) and 18.2 months (95% CI: 13.0–25.7) was observed in PTEN retained and PTEN loss patients, respectively (p = .71). PTEN loss patients were also found to have amplifications of EGFR gene more frequently than patients with retained PTEN (70.8% *vs.* 47.8%, p = .01). Multivariate analysis showed that older age (HR 1.64, CI: 1.02–2.63, p = .04), low KPS (HR 3.57, CI: 2.20–5.79, p<.0001), and *lack of standard* treatment (HR 3.98, CI: 2.38–6.65, p<.0001) yielded worse survival. PTEN loss was not prognostic of overall survival (HR 1.31, CI: 0.85–2.03, p = .22).

**Conclusions:**

Loss of expression of PTEN does not confer poor overall survival in the temozolomide era. These findings imply a complex and non-linear molecular relationship between PTEN, its regulators and effectors in the tumorigenesis of glioblastoma. Additionally, there is evidence that temozolomide may be more effective in eradicating GBM cancer cells with PTEN loss and hence, level the outcomes between the PTEN retained and loss groups.

## Introduction

Glioblastoma multiforme is the most common primary malignant brain tumor in adults with an average survival time of just over one year [1]–[7]. Even after standard treatment, which includes surgery followed by concurrent temozolomide and radiation (*i.e. Stupp* protocol), survival is only extended by an average of 2.5 months [1], [4], [8]. As standard medical practice yields little survival benefit, greater attention is being paid to personalized treatment and correspondingly to the expression of specific molecular markers with a goal to assess their possible therapeutic as well as prognostic significance [1], [5], [9]–[11]. Tyrosine kinase signal transduction pathways, especially epidermal growth factor receptor (EGFR) driven proliferation play an integral role in the pathogenesis of gliomas [1], [12]. PTEN is a tumor suppressor gene involved in several signaling pathways, most importantly the PI3K/Akt pathway in which it serves as a phosphatase acting on PIP3, dephosphorylating PIP3 and producing PIP2-a molecule that maintains inactivity in the Akt pathway [10], [13], [14]. PIP3 is a key regulator of the PI3K/Akt signaling pathway: it recruits Akt to the membrane surface, an event critical for Akt activation. Notably, the Akt pathway may be activated via binding of ligands to cell surface receptors, such as EGFR. Activated Akt regulates several downstream pathways controlling progression through the cell cycle, protein synthesis, survival, apoptosis and migration [15]–[17]. Loss of PTEN expression, through deletion, mutation or methylation essentially mimics activation of the Akt pathway as a result of the accumulation of PIP3 [15], while retention of PTEN maintains Akt inactivity [17], [18]. Although PTEN expression is ubiquitous across all tissues, only in certain tumor types has it been shown to play a role in tumorigenesis – such as tumors of the breast, ovaries, prostate, pancreas, skin, and most notably, brain [19]–[28].

PTEN-dependent dysregulation of signaling is very frequent in GBM, with mutation occurring in between 5% and 40% of all GBM cases, and loss of heterozygosity (LOH) in 60% to 80% of all cases [8]. Studies conducted in the pre-temozolomide era have demonstrated that loss of PTEN, either through loss of heterozygosity (LOH), methylation or mutation, had prognostic significance [29]. Alternatively, evaluation of cellular PTEN levels through expression profiling platform as well by immunohistochemistry have been found to be of prognostic significance in several other studies. This study aims to determine whether loss of PTEN still holds prognostic significance in newly diagnosed GBM treated in the temozolomide era.

## Materials and Methods

### Data Source

A total of 188 patients undergoing craniotomy for resection of a GBM between 2007 and 2010 were identified. Of these, 33 patients were excluded due to loss to follow-up, incomplete data or surgery related mortality (30-day post-op mortality). All patients underwent surgical resection of tumor (biopsy, partial resection, near gross total resection (≥95% resection) and gross total resection (all enhancing portions of tumor resected). Tumor grading was based on the WHO classification. This study was approved by the Institutional Review Board (IRB) of Cedars-Sinai Medical Center. Due to the retrospective nature of this study, the IRB approved a waiver of consent and therefore, consent was not needed (or obtained) for this study.

### Prognostic Variables

Patient age, gender, tumor location, post-operative Karnofsky Performance Scale (KPS), extent of resection, PTEN expression through IHC and EGFR amplification status as revealed by Fluorescence in-situ hybridization (FISH), treatment protocol and overall survival data was collected. For analysis, a patient's age was stratified into ≤65 or greater than 65, extent of resection was considered high if greater than 95% of the enhancing tumor was resected (*i.e.* near gross total resection, gross total resection), otherwise it was considered low (*i.e.* biopsy, partial resection). KPS scales were divided into scores reflecting normal function (KPS≥80) and those demonstrating inability to carry out normal function (KPS<80). Overall survival in months was calculated from the date of initial surgery and the social security death index was used to verify all date of deaths. PTEN expression was defined as loss or retained while EGFR expression was deemed normal or abnormal (*i.e.* amplification or polysomy).

### PTEN & EGFR analysis

Evaluation of PTEN staining was performed using immunohistochemistry. Four *µm* thick paraffin sections were deparaffinized through xylene, rehydrated by graded alcohol and pretreated with High pH buffer on Leica Bond-III automated stainer station. Subsequently, a commercially available antibody to PTEN (Cascade Biosciences ABM-2052) was used at 1∶250 dilution for 32 min incubation at room temperature followed by a detection with Leica Bond Refine DAB detection system. Nuclei were counterstained with Hematoxylin (Biocare Medical). A negative control was performed by omitting the primary antibody and this revealed no immunoreactivity.

**Figure 1:**
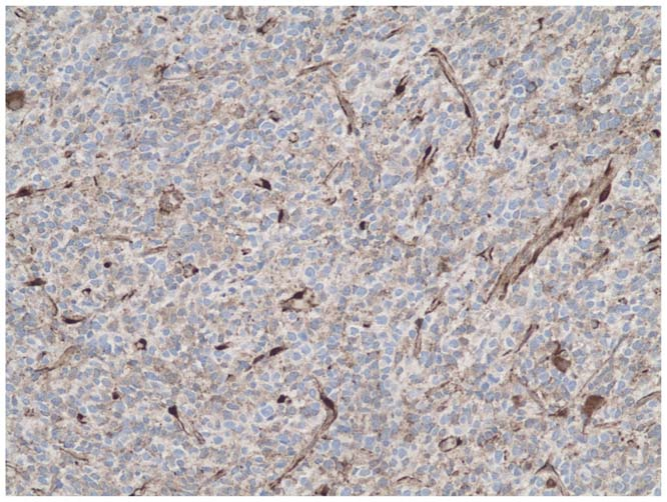
Immunohistochemical analysis, example of a patient with PTEN loss.

**Figure 2:**
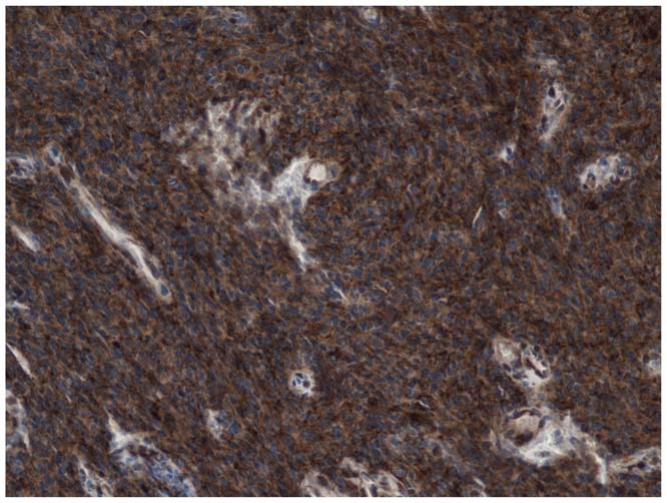
Immunohistochemical analysis, example of a patient with PTEN retained.

Evaluation of PTEN immunostaining was performed in at least 3 separate fields of the tumor, each containing not less than 50 tumor cells. Endothelial cells within the tumor were used as a positive control with a rank of staining assigned as 3+. Negative or weak staining (0+ or 1+) was considered as a loss of expression (**Figure 1**). A moderate, uniform cytoplasmic staining of tumor cells, to a degree lower than of endothelial cells was assigned 2+ intensity and considered to be preserved (**Figure 2**). Variation from one field to another was generally low in the most cases of GBM.

Evaluation of EGFR amplification by FISH was done using commercially available kit (Vysis, Downers Grove, IL). Dual-probe analysis was performed with a locus-specific probe for both EGFR, (the gene is located on chromosome 7), and for chromosome 7 with chromosome enumeration probe 7 (CEP7). The tumors with an EGFR/CEP7 signal ratio >2.00 were classified as having EGFR amplification. Tumors with greater than 10% nuclei with 3 or more CEP7 signals were classified as having gain of chromosome 7. Both gain of chromosome 7 and EGFR amplification frequently occur together in gliomas.

### Statistical Analysis

Overall survival was calculated from the time of surgery to death or time of last follow-up appointment for surviving patients. Chi-square test was used to evaluate potential differences in median overall survival by PTEN expression as well as patient baseline characteristics. Differences in overall survival by PTEN expression were evaluated via Kaplan-Meier estimates (log-rank test). Multivariate Cox proportional hazards regression models were used to explore the predictive role of patient and tumor characteristics (*e.g.* KPS, age, PTEN status) in overall survival.

## Results

A total of 155 newly diagnosed GBM patients were included in the study. Median age was 63 years, 64.5% were male and 98.1% had supratentorial tumors (**Table 1**). Independent functional level (KPS≥80) was documented in 69.7% of patients, 52.3% had gross total or near gross total resection and 80.7% were treated with standard therapy (radiation therapy with concurrent temozolomide), also known as the *Stupp* protocol [11]. While no significant associations were found between patient characteristics and PTEN expression levels, PTEN loss patients were generally older (53.0% *vs.* 44.4, p = .10), had higher rates of disability (4.8% *vs.* 1.4, p = .36), and had lower resection (43.1% *vs.* 51.9%, p = .28).

**Table 1:** Patient and tumor characteristics stratified by PTEN expression (retained vs. loss).

Characteristics	All patients [N (%)]	Retained [N (%)]	Loss [N (%)]	p value
Total number of patients	155	72	83	
Age				0.10
18–49	26 (16.8)	17 (23.6)	9 (10.8)	
50–64	53 (34.2)	23 (31.9)	30 (36.1)	
≥65	76 (49.0)	32 (44.4)	44 (53.0)	
Gender				0.60
Female	55 (35.5)	24 (33.3)	31 (37.4)	
Male	100 (64.5)	48 (66.7)	52 (62.7)	
Tumor Location				0.21
Infratentorial	2 (1.3)	2 (2.8)	0 (0)	
Supratentorial	152 (98.1)	70 (97.2)	82 (98.8)	
Both	1 (.7)	0 (0)	1 (1.2)	
Tumor Size in centimeters				0.20
1–3	19 (17.0)	8 (42.1)	11 (57.9)	
3–4	19 (17.0)	12 (63.2)	7 (36.8)	
4–6	48 42.9)	17 (35.4)	31 (64.6)	
≥6	26 (23.2)	13 (50.0)	13 (50.0)	
Karnofsky Performance Scale			0.36	
80–100 (normal activity)	108 (69.7)	49 (68.1)	59 (71.1)	
50–70 (requires assistance)	42 (27.1)	22 (30.6)	20 (24.1)	
≤40 (disabled)	5 (3.2)	1 (1.4)	4 (4.8)	
Extent of Resection*				0.28
Low	73 (47.7)	31 (43.1)	42 (51.9)	
High	80 (52.3)	41 (56.4)	39 (48.2)	
Standard Treatment**				0.66
Yes	125 (80.7)	57 (79.2)	68 (81.9)	
No	30 (19.3)	15 (20.8)	15 (18.1)	

* Near-gross/gross-total-resection high resection; biopsy/partial low resection.

** Standard treatment: radiotherapy+temolozolomide.

**Figure 3:**
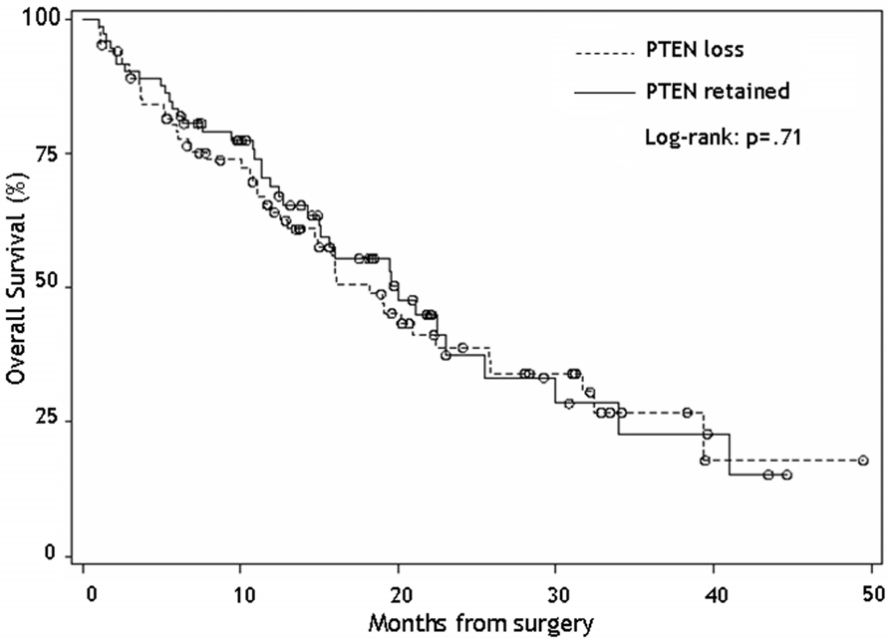
Kaplan-Meir overall 260 survival functions for newly diagnosed glioblastoma patients according to PTEN expression (dashed: PTEN loss, solid: PTEN retained).

Differences in median overall survival (OS) by patient characteristics were also evaluated. Older (≥65), higher functioning (KPS≥80), high extent of resection, and *Stupp* treatment were all statistically significant predictors of longer survival (**Table 2**). A total of 72 patients (47%) had retained expression for the PTEN gene. Independent of PTEN, the median OS was 19.1 months (95% CI: 15.0–22.5). Differences in median OS survival among PTEN retained versus loss (20.0 *vs.* 18.2 months, p = .71) patients were not statistically significant (**Table 2** **,** **Figure 3**). EGFR alterations were more common in PTEN loss patients (70.8% *vs.* 47.8%, p = .01). Patients with EGFR amplification and PTEN loss had a slightly worse median overall survival compared to patients with normal EGFR and retained PTEN (20.2 *vs.* 19.5 months, p = .55).

**Table 2:** Median overall survival by patient and tumor characteristics.

Characteristics	N	%	median overall survival in months	p value
Age				0.0005
18–49	26	16.8	41	
50–64	53	34.2	19.5	
≥65	76	49	15	
Gender				0.27
Female	55	35.5	25.5	
Male	100	64.5	16	
Tumor Location				-
Infratentorial	2	1.3	-	
Supratentorial	152	98.1	19.1	
Both	1	0.7	-	
Karnofsky Performance Scale			<.0001	
80–100 (normal activity)	108	69.7	25.7	
50–70 (requires assistance)	42	27.1	9.8	
≤40 (disabled)	5	3.2	6.3	
Extent of Resection*				0.01
Biopsy	40	26.5	15	
partial resection	33	21.9	11.5	
near gross total resection	22	14.6	19	
gross total resection	56	37.1	22.5	
EGFR Expression				0.25
normal	43	38.7	15.8	
abnormal	68	61.3	19.5	
PTEN Expression				0.71
loss	83	53.6	18.2	
Retained	72	46.5	20	
Standard Treatment**				<.0001
Yes	125	80.7	22.5	
No	30	19.3	5.4	

* Near-gross/gross-total-resection high resection; biopsy/partial low resection.

** Standard treatment: radiotherapy+ temolozolomide.

Multivariate analysis showed that older age (HR 1.64, CI: 1.02–2.63, p = .04), low functioning scores (HR 3.57, CI: 2.20–5.79, p<.0001), and lack of standard treatment (HR 3.98, CI: 2.38–6.65, p<.0001) were strong predictors of poor survival. PTEN loss did not significantly increase mortality hazards in the multivariate model (HR 1.31, CI: 0.85–2.03, p = .22) (**Table 3**).

**Table 3:** Multivariate analysis of Overall Survival: Adjusted hazards ratio (HR) of mortality, 95% Confidence Intervals (CI), and p values.

Characteristics	HR	95% CI	p value
Age in years (ref: ≤65)			
age>65	1.64	1.02–2.63	0.04
Karnofsky Performance Score (ref: KPS≥80)			
KPS<80	3.57	2.20–5.79	<.0001
Low vs. high extent of resection	1.39	0.89–2.17	0.14
Lack of Standard Treatment	3.98	2.38–6.65	<.0001
PTEN loss	1.31	0.85–2.03	0.22

## Discussion

Our study found that PTEN loss was not associated with worse overall survival in newly diagnosed GBM patients of the temozolomide era. Older age (≥65), dependent functional level (KPS<80), partial resection, and lack of *standard* therapy were significant predictors of poor overall survival in newly diagnosed GBM patients.

PTEN is a tumor suppressor gene that codes for a phosphatase that acts on PIP3 and regulates the PI3k/Akt pathway [17], [30]. Several previous studies, most of which were conducted in the pre-temozolomide era, demonstrated an adverse prognostic significance of PTEN loss of function. For instance, patients with loss of function mutations of PTEN generally had shorter survival than patients with PTEN retention [8], [29], [31]–[35]. Srividya et al. reported that 10q23/PTEN homozygous deletion was shown to be a significant predictor of poor survival (p = .027) in GBM patients, with average survival in the non-deletion cohort of 24 months, and average survival in the homozygous deletion cohort of 14 months. Furthermore, stepwise selection model demonstrated independent prognostic value of homozygous PTEN deletion (p = .033, HR: 2.013) [8]. Differences in the findings between Srividya et al. [8] and our study may be explained by the following factors. Their study included a smaller patient cohort of 73 glioblastoma patients and inclusion was restricted to patients who underwent either near gross or gross total resection (no patients had partial resection or biopsy). Furthermore, all patients had post-operative KPS scores of above 70, as opposed to our study that included patients with a wide range of KPS scores. Finally, the only variables included in Srividya et. al [8] study's multivariate analysis were patient age and PTEN status. Our study included all major previously confirmed prognostic factors in a more representative and much larger group of newly diagnosed GBM patients treated in the temozolomide era. These factors make our analysis more representative of all newly diagnosed GBM patients.

Temozolomide is a chemotherapeutic alkylating agent that can penetrate the blood brain barrier, making it much more effective in treating brain tumors than previously used chemotherapies [36]. There is evidence that temozolomide may be more effective in eradicating GBM cancer cells with PTEN loss and hence, level the outcomes between the PTEN retained and PTEN loss groups. McEllin et al. [37] reported sensitivity of human glioma cell lines with PTEN loss to temozolomide. They found that PTEN deficient glioma cell lines were inefficient at repairing double-strand breaks induced by Temozolomide, which lead to more apoptosis. This would explain why PTEN loss does not predict poor survival in GBM patients treated with temozolomide. Alternatively, other signaling molecules may circumvent PTEN retention and activate the PI3k/AKT pathway thus, nullifying the independent prognostic effects as discussed below.

**Figure 4:**
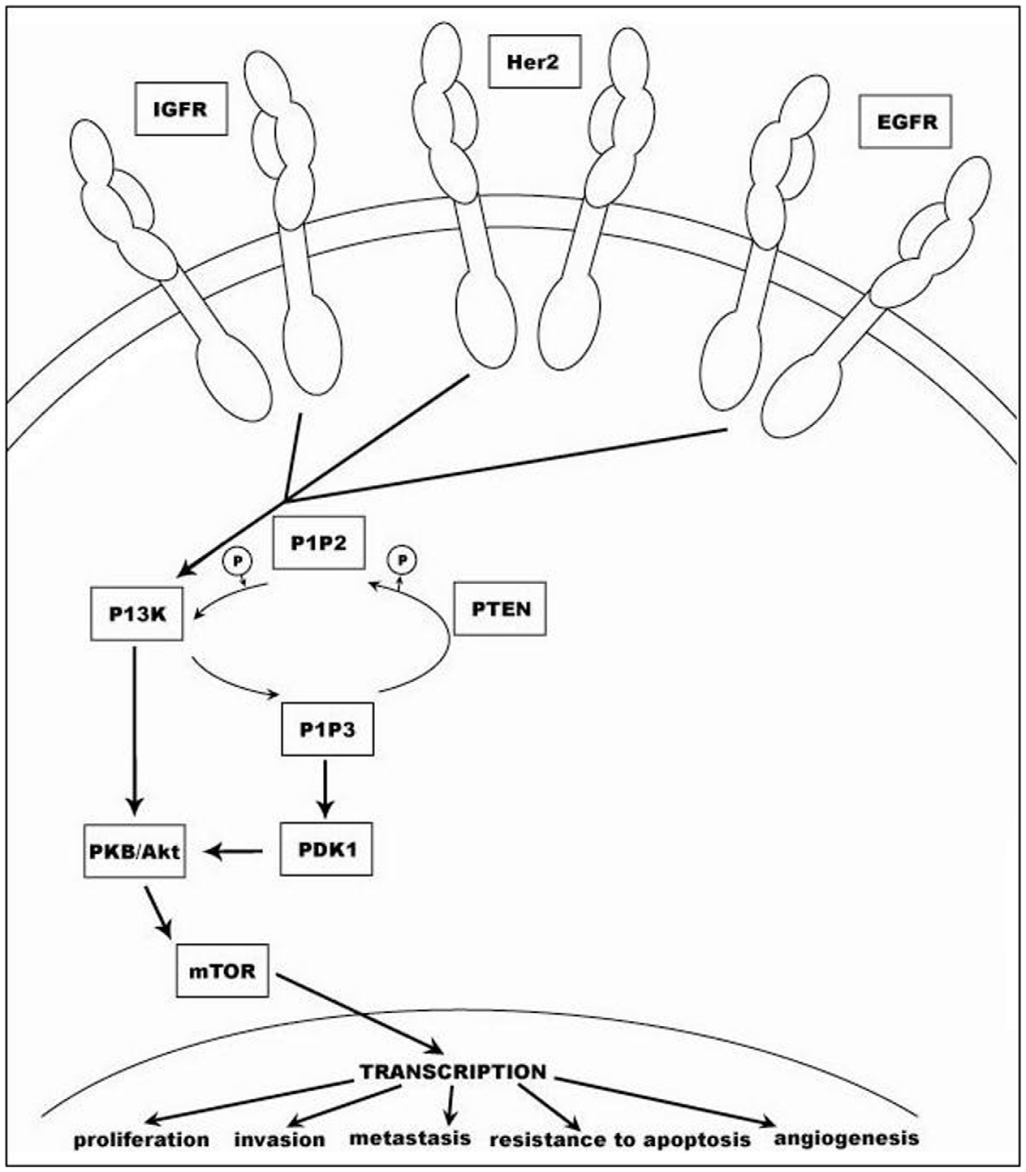
Role of PTEN in the PI3K-AKT signaling pathway.

PTEN, a protein and lipid phosphatase, is an important player in the PI3k/Akt signaling transduction pathway, a pathway that regulates critical cell functions such as progression through the cell cycle, cellular differentiation, migration, and most importantly, apoptosis (**Figure 4**) [30], [38]. The pathway is initially activated by the binding of a ligand to a receptor on the plasma membrane, such as EGFR, HER2 or IGFR, triggering the subsequent activation of PI3k (also known as phosphatidylinositol 3-kinase) – whose main function is to phosphorylate the 3′ hydroxyl group of the inoisitol ring of phosphatidylinositol [38]. The product of PI3k, a molecule called PIP3, acts as the main substrate for PTEN. PIP3 plays an integral role in recruiting Akt to the surface of the membrane, an event critical for the activation of the PI3k/Akt pathway. PIP3, a membrane bound protein, triggers PDK1 activation and subsequent Akt phosphorylation and recruitment to the membrane and it is this Akt activation that directly regulates all functions of the PI3k pathway. The role of PTEN is to dephosphorylate PIP3 into PIP2, rendering it incapable of recruiting Akt to the surface of the membrane and thus activating the PI3k/Akt pathway (**Figure 4**). Loss of PTEN results in accumulation of PIP3. This constitutively activates the PI3k/Akt pathway inducing tumorigenesis through cell cycle progression, invasion and loss of apoptosis.

The PI3k/Akt pathway can be up-regulated due to mutation such as deletion and translocation in one or more of its downstream effectors [29], [39]. While previous literature has advocated the independent prognostic significance of PTEN loss in glioblastoma [1], [8], [29], [31], other studies have demonstrated the importance of EGFR amplification as well as other methods of Akt pathway activation in tumorigenesis [1], [40]–[42]. In their landmark paper, Mellinghoff et al. utilized the same immunohistochemistry analysis of PTEN as the current investigation to demonstrate that EFFR kinase inhibitors were only effective in patients who had retained PTEN and an EGFviii mutation [43]. Our study found a correlation between PTEN loss and EGFR amplification. It has been reported that synthetic amplification of EGFR in heterozygous PTEN knockout causes mice to develop invasive brain tumors that closely resemble human glioblastomas [1]. Amplification or loss of regulation of several other molecular markers in addition to EGFR and PTEN involved in the PI3k pathway have been implicated in human glioblastoma: platelet-derived growth factor receptor (PDGFR), fibroblast growth factor receptor (FGFR), and other tyrosine kinase receptors [1], [12], [21]. Other studies have demonstrated the significance of yet other effectors in the Akt pathway–whose mutation results in up-regulation of the pathway [1], [7], [15], [44]–[47].

While PTEN expression versus retention does not independently demonstrate prognostic significance, other molecules involved in the PI3k/Akt pathway could hold independent or correlative prognostic significance. For example, although Pelloski et al. [48] found no significant association between activated Akt and overall survival, their univariate results did show decreased survival associated with other members of the Akt pathway (p70S6K, mTOR). However, that effect was not significant in their final multivariate model. Pelloski et al. also attempted to associate the loss of PTEN to Akt expression in 142 patients with glioblastoma and found that there was no inverse correlation between PTEN and Akt as assessed by immunohistochemistry. Other large studies in breast cancer and melanoma have also failed to detect the inverse relationship between PTEN and Akt and some have even reported an unexpected positive correlation [36], [49]. This can be accounted for by the fact that several other proteins in addition to PTEN are involved in Akt activation and its recruitment to the membrane—such as PDK1, insulin and insulin-like growth factor-1, which are responsible for phosphorylating, and thus activating Akt [15], [44], [45], [50]. For example, if PDK1 constitutively activates Akt, thus activating the PI3k pathway, Akt activation occurs independently of PTEN loss or retention, resulting in PTEN-independent constitutive activation of the PI3k/Akt pathway. Furthermore, there are other direct methods of Akt activation not involving PTEN: modifications to Akt itself –such as regions of the protein involved in membrane recruitment and also resulting in constitutive Akt activation independent of PTEN status [44], [45], [50]. The fact that an inverse correlation between PTEN and Akt was not found, in a large number of GBM samples, supports the idea of a complex relationship between PTEN, Akt and their regulators and effectors.

Our study evaluating the role of PTEN as a prognostic marker in the temozolomide era included the largest, most representative group of newly diagnosed GBM patients. After controlling for all key prognostic factors we found that PTEN status alone did not hold independent prognostic significance and did not predict overall survival. These findings imply a complex molecular relationship between PTEN and its regulators and effectors in the tumorigenesis of glioblastoma. Additionally, there is evidence that temozolomide may be preferentially effective in eradicating GBM cancer cells with PTEN loss and hence, level the outcomes between the PTEN retained and PTEN loss groups.
